# Quantifying the effect of *Jacobiasca lybica* pest on vineyards with UAVs by combining geometric and computer vision techniques

**DOI:** 10.1371/journal.pone.0215521

**Published:** 2019-04-22

**Authors:** Ana del-Campo-Sanchez, Rocio Ballesteros, David Hernandez-Lopez, J. Fernando Ortega, Miguel A. Moreno, on behalf of Agroforestry and Cartography Precision Research Group

**Affiliations:** Agroforestry and Cartography Precision Research Group, Institute for Regional Development, University of Castilla—La Mancha, Albacete, Spain; Wageningen Universiteit en Research, NETHERLANDS

## Abstract

With the increasing competitiveness in the vine market, coupled with the increasing need for sustainable use of resources, strategies for improving farm management are essential. One such effective strategy is the implementation of precision agriculture techniques. Using photogrammetric techniques, the digitalization of farms based on images acquired from unmanned aerial vehicles (UAVs) provides information that can assist in the improvement of farm management and decision-making processes. The objective of the present work is to quantify the impact of the pest *Jacobiasca lybica* on vineyards and to develop representative cartography of the severity of the infestation. To accomplish this work, computational vision algorithms based on an ANN (artificial neural network) combined with geometric techniques were applied to geomatic products using consumer-grade cameras in the visible spectra. The results showed that the combination of geometric and computational vision techniques with geomatic products generated from conventional RGB (red, green, blue) images improved image segmentation of the affected vegetation, healthy vegetation and ground. Thus, the proposed methodology using low-cost cameras is a more cost-effective application of UAVs compared with multispectral cameras. Moreover, the proposed method increases the accuracy of determining the impact of pests by eliminating the soil effects.

## Introduction

Viticulture is the cornerstone of many rural regions, and grapevines are one of the most important crops grown in France, Spain, Australia, South Africa, and parts of the USA, Chile and Argentina, among other countries. This crop is important not only because of its growth area but also due to its economic impact in rural areas. Therefore, improving crop management is essential for ensuring the sustainability of small holdings as well as the promotion of large wineries in the international market. In Spain, vineyards cover 931,065 ha, which represents 26.6% of the total vineyard surface area in Europe [1]. From 2009 to 2015, wine-producing vineyards in Spain have increased from 39,259,000 hl to 44,415,000 hl, [2]. The high amount of land dedicated to this crop and the progressive increase in production during recent years are the reasons why early detection of agronomic constraints from pests and diseases as well as fertilization and water requirements are some of the main aspects used to improve viticulture management.

The use of pesticides by crop area in the world from 1990 to 2014 has increased by an average of 4.47% per year [1]. Agriculture consumes approximately 95 million tons of fertilizer and 97,000 tons of pesticides and herbicides as active ingredients [1]. Efficient use of phytosanitary products, which occurs when only affected plants are treated, involves not only reducing costs but also improving sustainable management practices.

Infestation by the leafhopper *Jacobiasca lybica* is considered dangerous for vineyards. Adults overwinter on evergreen plants and infest grapevines in spring. Symptomatology occurs in summer in the form of leaf discoloration and even leaf drying if the attack is severe. The lack of photosynthetic activity results in an increase in soluble solid concentrations in its fruits, which in turn spoils the vine production (20% reduction in harvest and lower quality) [3]. The losses caused in the quantity and quality of the harvest has motivated this research to determine the optimal time to apply phytosanitary products, to determine the effects of this pest on current crops, and to identify any impacts on crops in the following season.

Traditional practices to control the potential impacts of this disease are typically based on field observations for identifying and quantifying infested plants. This work is often tedious and unaffordable when covering large areas. Instead, the use of precision agriculture techniques using remote sensing for the automation of pest monitoring is now a widely used approach [4–9].

Strong efforts have already been made in precision agriculture to generate remote sensing information through the use of satellite-based imagery. Nevertheless, the main constraints of these platforms are their low spatial and temporal resolution, which often involve a lack of information about crop health status because the quality of the pixel size or capture frequency are not high enough. If spatial resolution is low, each pixel collects heterogeneous surfaces (soil, vegetation, neighboring crops, shadows, etc.), so the analysis of that information must consider the lack of details about the crop. Low temporal resolution does not take into account relevant changes in the phenology stages; if the temporal frequency of the image capture does not consider the phenology stage of the crop, a crop cycle could be incomplete or the digitalized data could be useless.

The use of very-high resolution remote sensing by unmanned aerial vehicles (UAVs) is becoming one of the most promising tools for precision agriculture. Progress in the development of hardware and software has led to the widespread use of UAVs and ground sensors to notably increase temporal and spatial resolution. The image capturing process was planned for the proposed objective, so the spatial resolution is adequate for collecting sufficient details about the crop; therefore, this process can be executed precisely when it is needed in the vegetal cycle. These aspects are great advantages compared to low-cost satellite-based technologies, and UAVs are widely accepted as a novel form of technology. Indeed, the UAV market is exponentially increasing worldwide with more than 3,000 operators in the USA, more than 2,000 in France, and close to 1,500 in the United Kingdom [10]. In Spain, there were more than 3,000 operators in 2017. In addition, sensor miniaturization and the improvement of sensor accuracy are allowing this technology to be applied at a relatively low cost with the added benefits of higher temporal and spatial resolution [11–15]. The main limitation for this platform is the autonomy of the aircraft to cover a large area in a single flight. Nevertheless, the UAV market has solutions that can provide flights that cover more than 200 ha per flight, which is sufficient for addressing most agricultural problems. Fixed wing vehicles could even cover a larger area; however, in many countries, their use is limited due to legal issues. For agricultural applications, UAVs have shown potential as aerial platforms to monitor crops [11,16,17], determine plant height (growth) [18–22], map weeds among various crop types, such as agave, sunflower, maize, tomato, vineyards, and wheat [5,12,23–29], and many other applications. Additionally, some authors have focused their research on mapping alterations in the phytosanitary status of crops with UAVs [9,20,21,30,31] or other piloted aircraft [32–34]. Regarding pest mapping with RGB cameras, [35] noted that the use of RGB imagery taken from an UAV is more efficient than conventional visual assessments for estimating the resistance of potato plants to late blight.

Recently, [20] examined whether spectral, hyperspectral, canopy height and temperature information could be derived from handheld and UAV-borne sensors to discriminate between sugar beets cultivars that are susceptible or tolerant to beet cyst nematodes. In conclusion, these authors determined that the most valuable traits for this task, according to validity, were canopy height, spectrally inferred chlorophyll content, leaf area or biomass, and canopy temperature.

The literature on detecting pests with UAVs has focused on the use of planimetric (2D) geomatic products (RGB, thermal, multispectral or hyperspectral orthoimages) through vegetation indices [5,25,36–40] or computational vision processes [12,29,41,42]. However, using only 2D information could lead to inaccurate estimations of the impacts of pest when the radiometric response of affected plants is similar to the radiometric response of the soil, weeds, shadows, or elements near the plants. This problem could be solved if vegetation could be precisely segmented from the soil using geometric techniques in a 3D (Three-dimensional) point cloud obtained using photogrammetry techniques. The combination of 3D and 2D treatments enhanced the accuracy of the generated results. [43] performed ground segmentation on detailed orthoimages based on the differences in the colors between the vegetation and soil. In determining the impact of *J*. *lybica*, leaves could be miss-classified, i.e., affected green leaves that are turning brown could be confused with soil. As affected leaves are always located at a higher level than soil, the combination of computer vision techniques (2D crop information) and geometric (3D crop information) using a 3D model of the crop would allow for these pixels to be segmented.

Thus, the main objective of this paper was to develop a methodology that combined geometric and computer vision techniques for quantifying the impact of *J*. *lybica* on a parcel of vines supported by a trellis using radiometric and tri-dimensional information generated by RGB cameras mounted on UAVs.

## Materials and methods

### The case study

**Fig 1:**
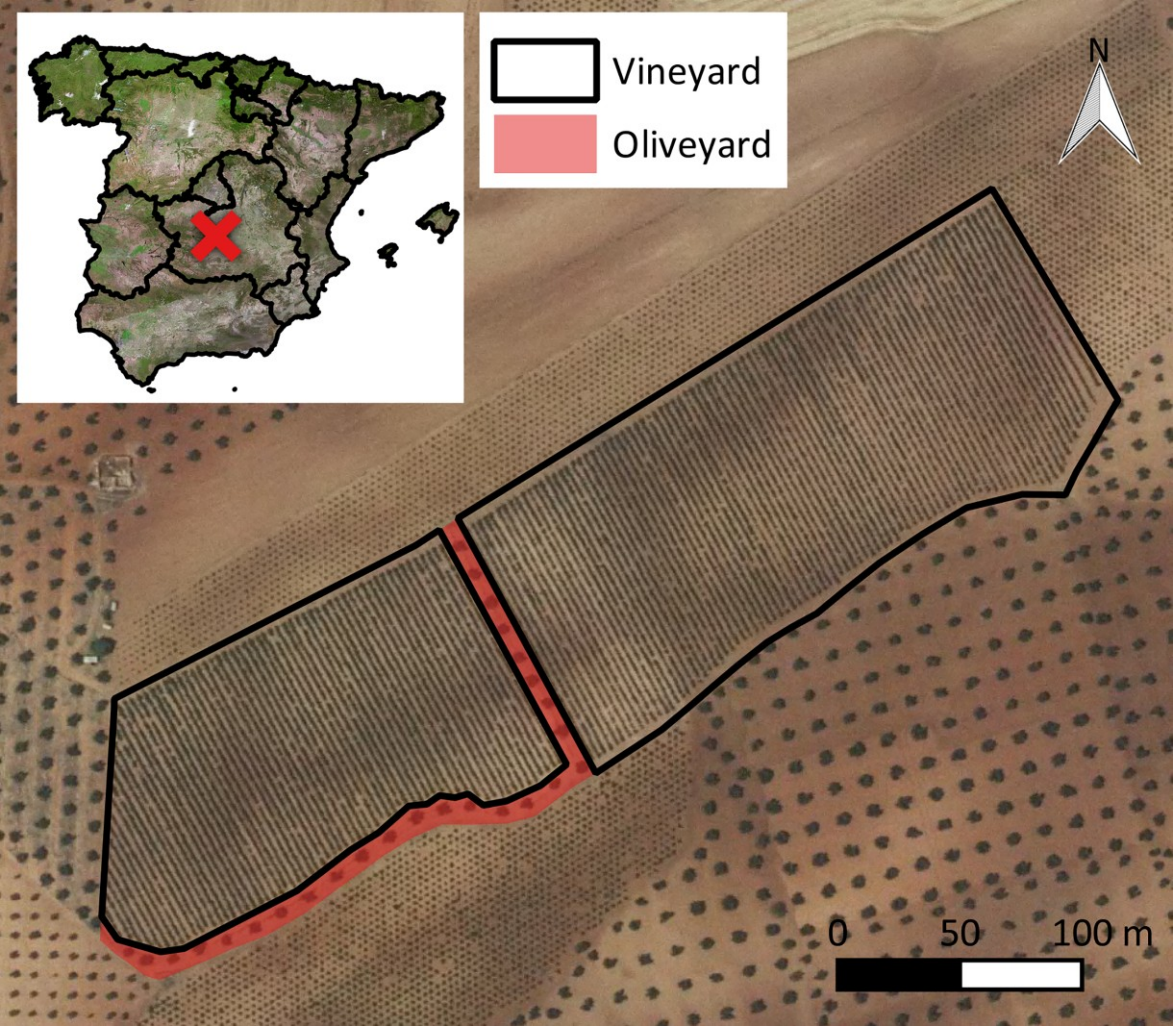
Location of the case study plot. Source: Spanish National Plan of Aerial Orthophotography.

An irrigated commercial vineyard was considered in this study: *Vitis vinifera* L. cv. *Sirah*. The owner of the land gave permission to conduct the study on this site. The training system was comprised of a trellis (four vertical wires). This vineyard was located in Madridejos, Spain (Fig 1), at 39.406834°, -3.579190° (EPSG: 4326, European Petroleum Survey Group). The plot area was 5.03 ha (4.71 ha wine crop and 0.32 ha olive oil crop) (Fig 1). The crop was affected by the *J*. *lybica* pest around mid-August 2016. Pesticides were not applied immediately after pest detection. A farmer applied the product on the 2^nd^ of September (one week before flight performance). The farmer’s main interests regarding UAV surveillance focused on 1) quantifying the affected area for insurance purposes and 2) locating areas where an additional treatment could be applied to decrease the impact on the following season. For both goals, an accurate thematic map that accounts for the effects of pests was required.

### Equipment

**Fig 2:**
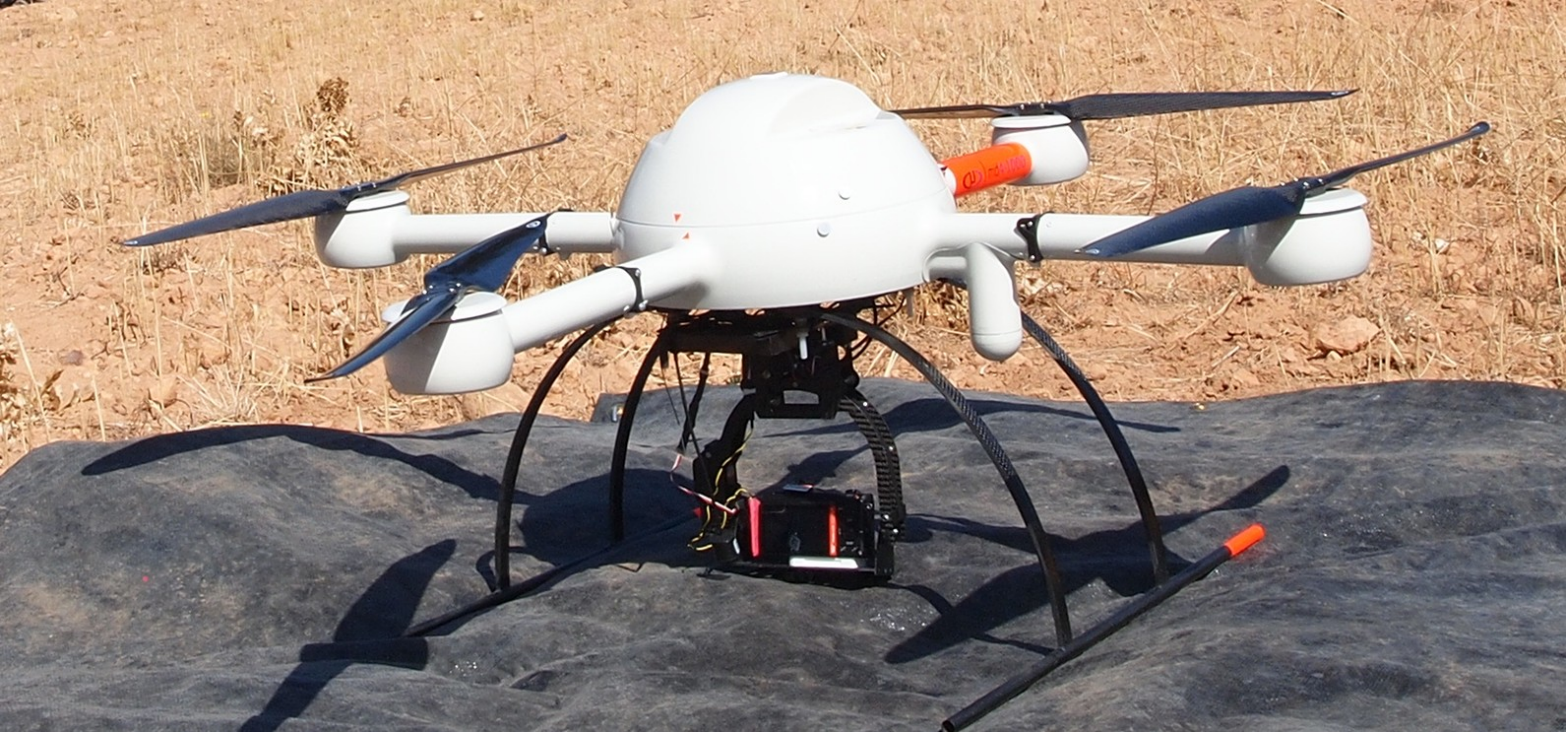
UAV utilizing a mounted SONY α ILCE-5100L sensor + E 20 mm F2.8 lens.

The UAV used for the photogrammetric flight was a microdrone md4-1000 (Microdrones, Inc., Kreuztal, Germany). The sensor was a SONY α ILCE-5100L with a E 20 mm F2.8 lens (SONY Corporation, Tokyo, Japan). The main characteristics of the UAV flight and the sensor capture mode and setting are listed in Table 1. Fig 2 shows the UAV and camera.

**Table 1:** Main characteristics of the UAV and sensor settings during the flight.

UAV microdrone md4-1000	SONY α ILCE-5100L + E 20 mm F2.8
Vertical speed: 1.0 m s^-1^ Cruising speed: 5.0 m s^-1^ Flight length: 2 km Flight height: 80 m Flight time: 10 minutes	Weight: 238+69 g Sensor pixel size: 0.004 x 0.004 mm Image size: 6,000 x 4,000 pixel Focal Length: 20 mm Shutter speed: 1/1600 s ISO: 100 F-stop: F/3.5

## Methodology

**Fig 3:**
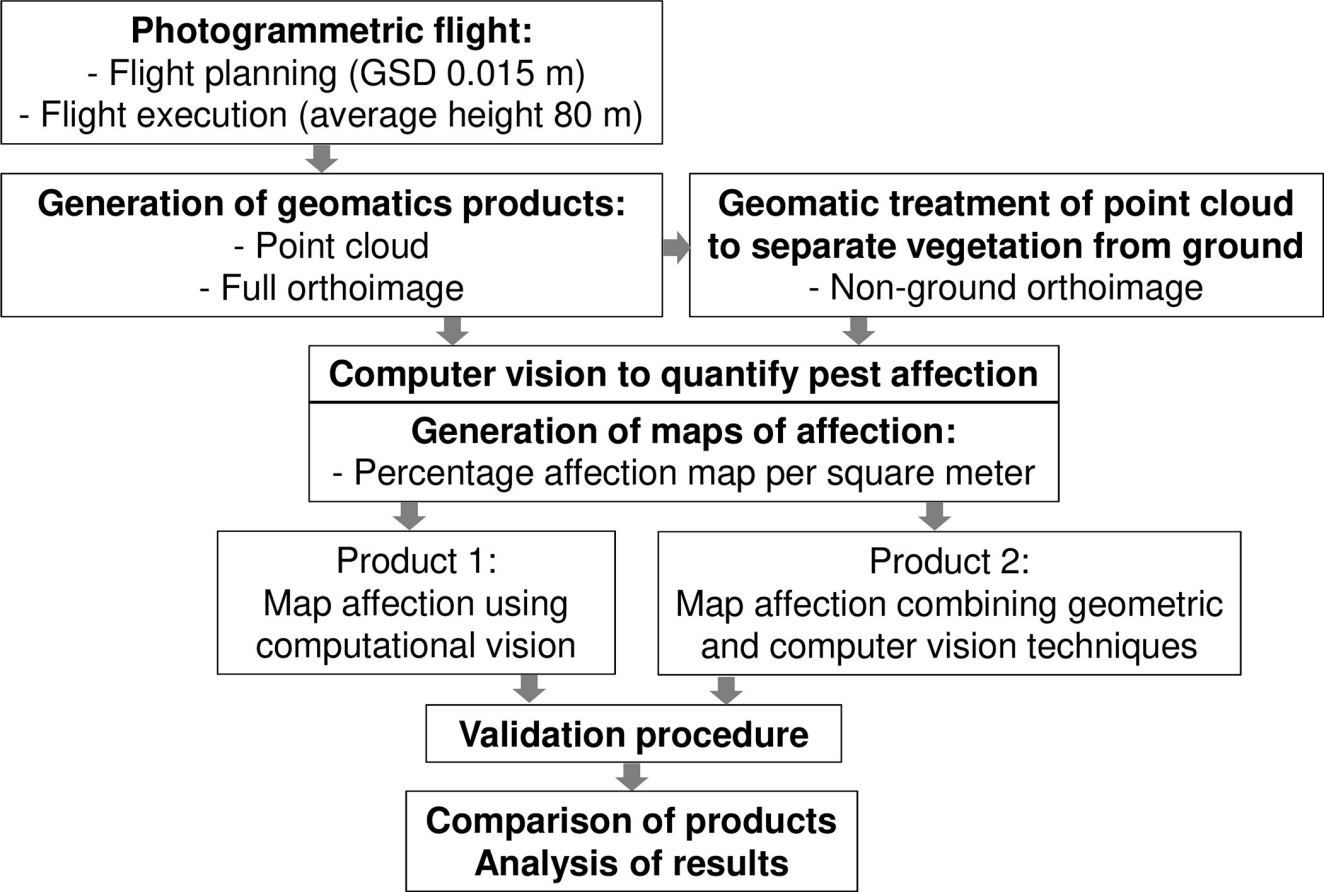
Flowchart of the proposed methodology. GSD: Ground Sample Distance.

The proposed methodology is summarized in Fig 3. A flight was planned based on overlapping values of 60% (forward) and 40% (side). The crop cover presents a convex shape that can be described covered by the overlapping values [44]. An orthoimage was obtained using photogrammetry techniques. In addition, a dense point cloud was generated and segmented into vegetation and ground using geometric techniques, as described below. Following this step, other orthoimages were generated using the dense point cloud that only corresponded to vegetation. Both orthoimages were processed with computer vision techniques for segmenting pest impact pixels from healthy vegetation. The ground truth was obtained from the full orthoimage due to the high resolution of this product. Finally, the percentage of the affected surface was calculated and compared to determine the improvements in the proposed methodology.

### Data acquisition and photogrammetry process

**Fig 4:**
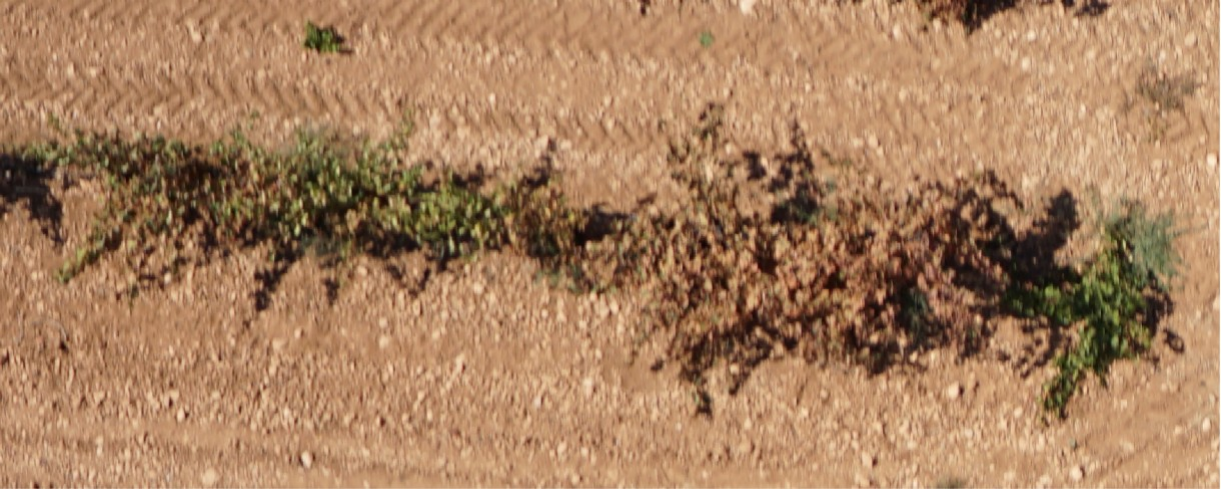
Appearance of healthy and affected leaves.

The flight was performed on September 9^th^, 2016. At this time, the berries were ripe for harvest. The healthy vines had not begun to exhibit leaf discoloration (senescence) at this phenological time. Thus, visual inspection was performed on the final high-resolution orthoimage to confirm the possible detection and location of the infested plants at that date, as shown in Fig 4.

**Fig 5:**
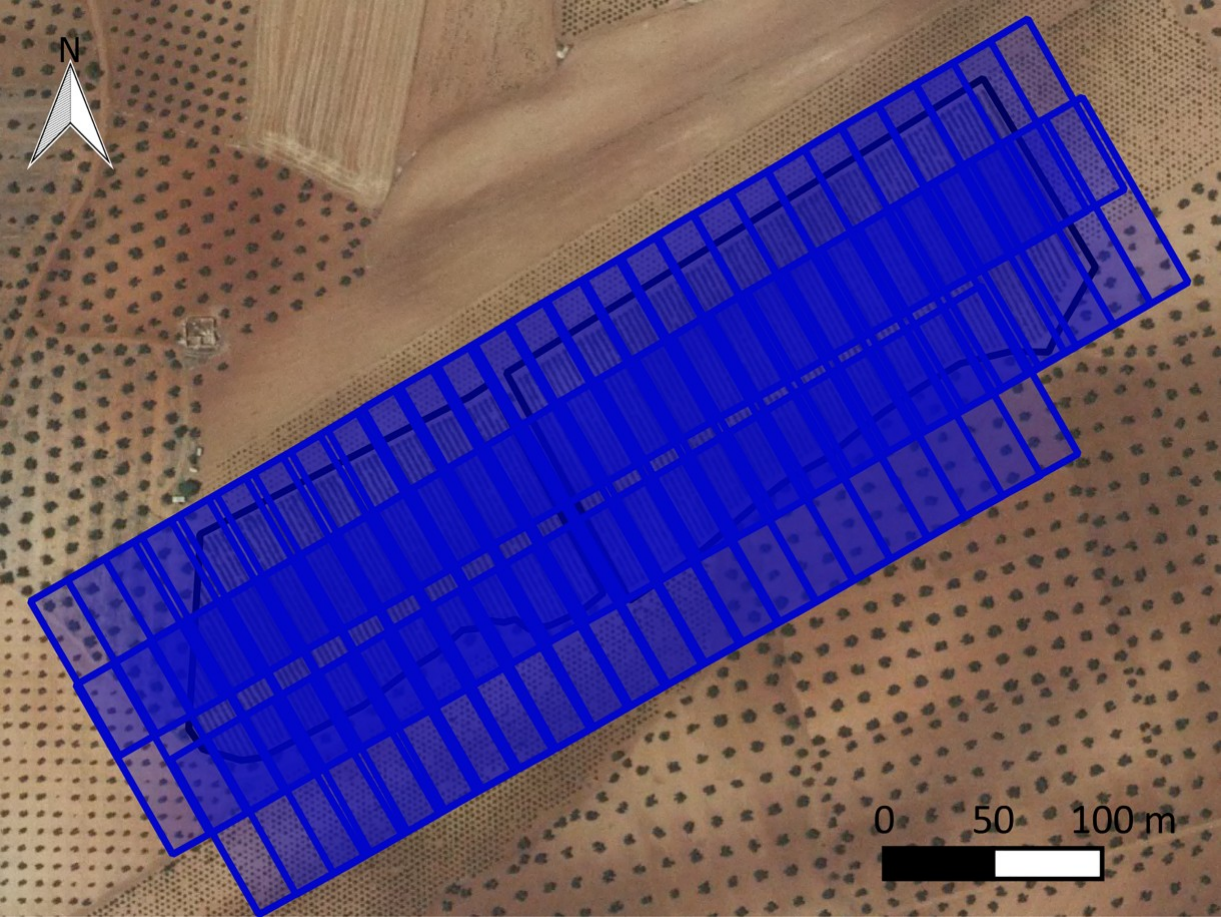
Footprint of the images for the flight planning process.

Flight planning was performed by using Microdrone Photogrammetric Flight Planning software (MFLIP) [45]. This software assures the correct overlapping values are used (60 and 40%, forward overlap and side overlap, respectively) because it accounts for GPS errors and camera angle precision among other sources of position errors. Furthermore, this software can incorporate accurate digital elevation models to fit the flight height to the terrain and, therefore, it can maintain a constant ground sample distance (GSD). A public digital elevation model (DEM) with 5 m spatial resolution that was freely provided by the National Geographic Institute of Spain was used for this propose. The main purpose of the flight planning process was to acquire the navigation file, which was then transferred to the UAV. In addition, this approach generated a database with the theoretical footprints of the images and the overlapping areas, among other data, in vector format to be examined with any GIS (Geographical Information System) software. The result of the flight planning was 165 images that were obtained with three strips (Fig 5).

The software Agisoft PhotoScan (Agisoft LLC, St. Petersburg, Russia), version 1.3.4, was used to perform the photogrammetric process. The main parameters that were considered to solve the photogrammetry process are listed in Table 2.

**Table 2:** Parameters used in the photogrammetric process.

**Point Cloud**	
Accuracy	High
Generic preselection	Yes
Key point limit	40,000
Tie point limit	4,000
Adaptive camera model fitting	Yes
**Dense Point Cloud**	
Quality	High
Depth filtering	Mild
**DEM**^**a**^	
Source data	Dense cloud
Interpolation	Enabled
**Orthomosaic**	
Blending mode	Mosaic
Surface	DEM
Enable color correction	No
Enable hole filling	Yes

^a^ Digital Elevation Model

To avoid the location of ground control points (GCPs) before performing the flight, the internal orientation of the camera was performed through previous calibration flights [46]. Moreover, to georeference the obtained orthoimage, seven natural features (e.g., fixed stones or path crosses) were used as GCPs. The coordinates of these GCPs were obtained using geomatic products that were freely supplied by the National Plan of Aerial Orthophotography (PNOA) (GSD = 0.5 m) (Spanish National Plan of Aerial Orthophotography 2015). The estimated georeferencing error following this methodology was approximately 0.5 m [46].

Vegetation and ground segmentation were performed for the point cloud using a tool in the Agisoft PhotoScan interface called Classify Ground Points. The geometric segmentation was based on two steps. The first step consisted of dividing the dense cloud into cells of a given size by the user. Next, a triangulation with the lowest points within the cropped cloud was calculated as the first approximation of the terrain model. The second step was an iterative process in which new points were included in the triangulation (and classified as ground), which satisfied the given parameters for the maximum angle and distance from terrain approximation. The utilized parameters included a maximum angle of 15°, a maximum distance of 1 m and a cell size of 6 m when taking into account the crop shape and ground rugosity of this parcel to segment ground from the vegetation. This geometric filtering performed well on vines attached to a trellis or isolated trees because changes in slope between the ground and the vegetation were abrupt. After segmenting the point cloud into vegetation and ground, one orthoimage was obtained using the classified vegetation points. The final geomatic products that were obtained were: 1) two orthoimages with a GSD of 0.015 m (full and nonground pixels), and 2) a dense point cloud with an average of 1,536 points m^-2^ that was segmented into vegetation and ground.

### Quantification of impact with computer vision techniques

**Fig 6:**
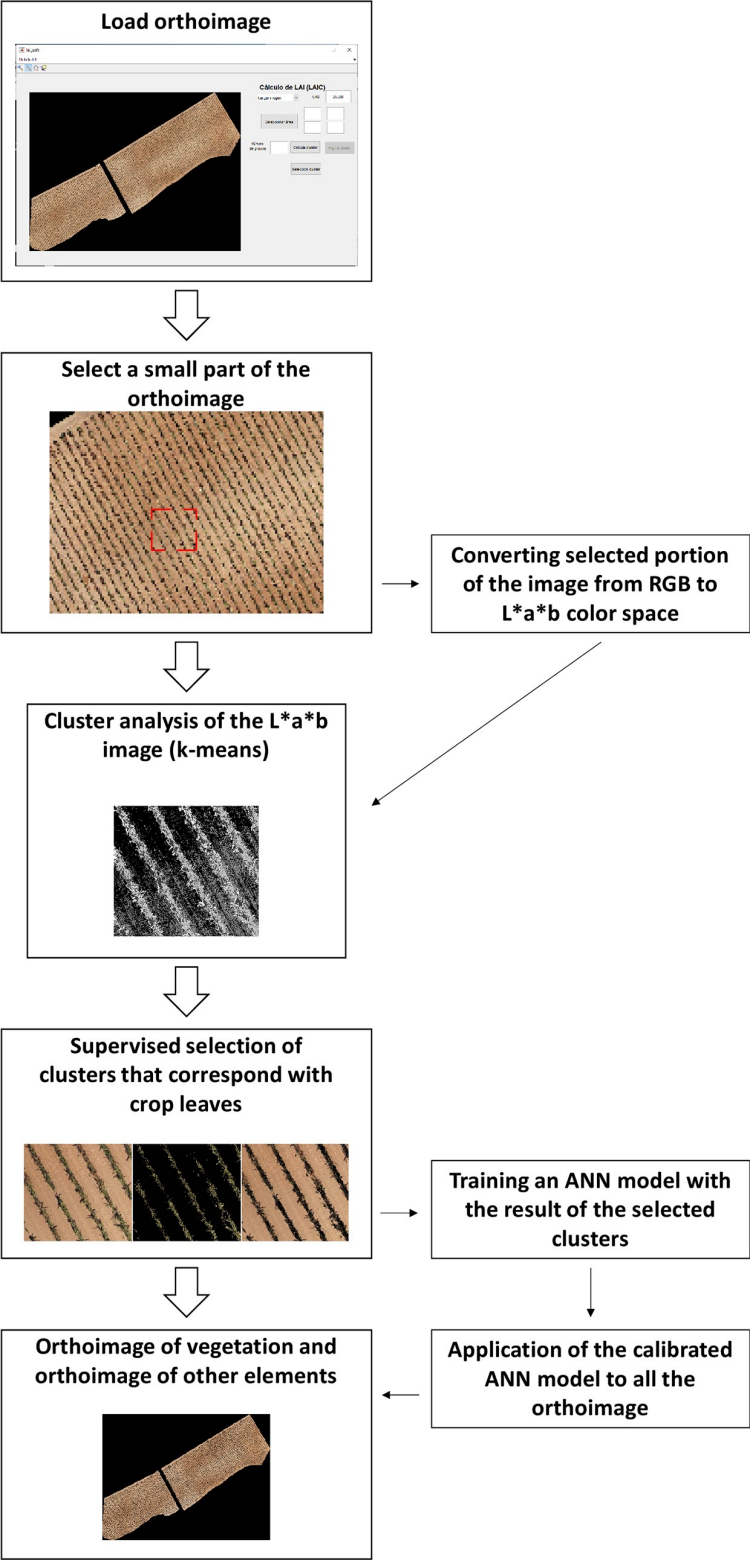
Flowchart of LAIC software.

The automated identification of affected vegetation was performed using the Leaf Area Index Calculation software (LAIC) [36]. This software was originally developed to discriminate green canopy cover from other features (ground, stones, and shadows, among others) in very high resolution aerial images. This software was also successfully applied to detect hydromorphological features in rivers [47,48]. The LAIC software (Fig 6) bases the classification on a supervised classification technique using Artificial Neural Networks (ANNs) [36]. In brief, the original orthoimage is loaded into the software, and a small part of the orthoimage is selected. For this small part of the orthoimage, the RGB color space is transformed into CIE-Lab color space (Commission Internationale de l’Eclairage–Lab) in which L is lightness, a is the green to red scale, and b is the blue to yellow scale. With a small part of the orthoimage transformed into the CIE-Lab color space, a cluster segmentation (k-means) was implemented using only the a and b components of this color space. Once the cluster segmentation was performed using clusters defined by the user between 2 and 10, the user should manually relate each cluster to the groups of features that appear in the image (e.g., ground, stones, healthy vegetation, affected vegetation, or shadows). Once the group of pixels identified by the user as affected vegetation are selected, these data are used to calibrate an ANN in which the input nodes correspond to the RGB values of each pixel, and the output node is 1 for affected vegetation and 0 for healthy vegetation and other features. After the ANN is calibrated with the small, treated part of the orthoimage, the calibrated ANN is applied to the remaining image. A raster was then created with assigned values of 1 and 0 for each pixel of the orthoimage in which 1 denoted affected vegetation and 0 indicated unaffected vegetation and other features.

Computer vision techniques using the LAIC software were applied to the original orthoimage in which the soil, affected and unaffected vegetation features appeared as well as in the orthoimage obtained after vegetation and soil segmentation.

### Final products and analysis of results

**Fig 7:**
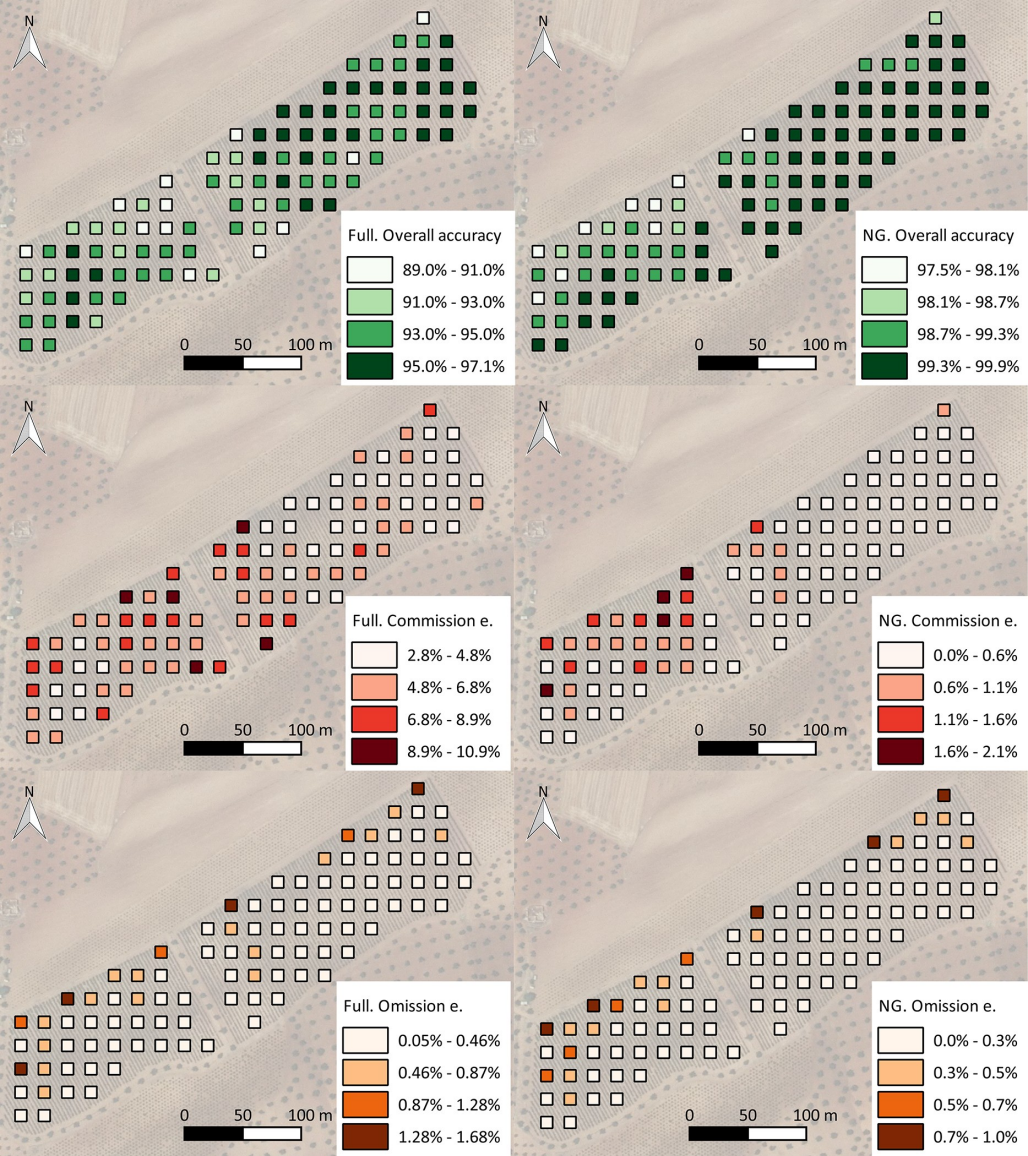
Orthoimage (left) and impact detected with computer vision techniques (right).

To evaluate the two classification methods, the ground truth information was obtained from the full orthoimage. A mesh of 97 squared polygons (10 m x 10 m) separated by 10 m was generated (Fig 7), which represents 20.6% of the total area. The detected effects of the pest inside these polygons were delimited manually by drawing as many irregular polygons as needed that represent affected pixels. Then, these digitalized areas were rasterized with the same pixel size and origin than the original orthoimages. This process resulted in a mask raster layer of the ground truth to be compared to the results of both methodologies evaluated in this work.

To assess the accuracy of both classification methodologies for the generated ground truth data, a confusion matrix was computed by considering the information pixel by pixel (Table 3). Overall accuracy (OA) (Eq 1) and success percentages (SP) (Eqs 2 and 3) were also computed. The OA indicates how well a certain area was classified by each methodology because it is the probability of a pixel being correctly detected by each methodology. Success percentages demonstrate how many pixels were correctly segmented.

**Table 3:** Confusion matrix and equations.

	Validation data		
Obtained data	Affected pixels	Non-affected pixels	Total pixels
**Affected pixels**	TP: True Positives for affection	FP: False Positives for affection	Total pixels affected
**Unaffected pixels**	FN: False Negatives for affection	TN: True Negatives for affection	Total unaffected pixels

$$OA(\%)=\frac{TP+TN}{TP+TN+FP+FN}$$ (1)

$$SPA(\%)=\frac{TP}{TP+FN}$$ (2)

$$SPN(\%)=\frac{TN}{TN+FP}$$ (3)

where OA is overall accuracy, SPA is success percentage of affection, and SPN is success percentage for no affection.

Additionally, to compare the results obtained with the use of computer vision and the integration of geometric and computer vision techniques, a mesh of 1 m^2^ was generated for the entire parcel. The average values of pixels with and without impact (1 or 0) were added and multiplied by 100 to produce a percentage of affected vegetation compared to unaffected vegetation. To analyze the results, the average percentage of impact of the entire plot was calculated. Additionally, thematic maps of impact were generated to evaluate the regions of the plots that experienced high and low impacts.

## Results and discussion

### Photogrammetric flight and preliminary geomatic products

All of the discolored leaves with brown tones caused by the aforementioned pest and not by other causes were detected. The observations that were carried out in the field and the visual inspection of the final orthoimage (i.e., Fig 4) made this identification possible. As indicated by Lentini et al. (2000), red grape cultivars affected by this pest change to a red color, but the level of infestation in this experiment was very high, and the leaves had already gone through this phase of discoloration. Desiccation was found in the next phase, and the dry leaves of these plants led to the loss of reddish coloration, which turned them brown.

The geomatic products obtained by photogrammetric techniques had a minimum GSD of 1.50 cm pixel^-1^. The tie points for the cloud characteristics are shown in Table 4.

**Table 4:** Detected tie points and generated dense point cloud.

**Tie Point Cloud**	
Points	159,939 of 178,838
RMS^a^ reprojection error^b^	0.450277 (1.45519 pix)
Max reprojection error	1.37839 (42.9695 pix)
Mean key point size^c^	3.41713 pix
**Dense Point Cloud**	
Points	144,892,166

^a^ Root Mean Squared

^b^ Refers to the distance between the point on the image where a reconstructed 3D point can be projected, and the original projection of that 3D point detected on the photo and used as a basis for the 3D point reconstruction procedure.

^c^ Mean tie point scale averaged across all projections.

Following the recommendations embodied in past experiences [11,16,45,46], the solution of the photogrammetric process was correctly concluded as expected. The conditions imposed in the flight plan to meet the geomatic proposed objectives when taking into account all technical, legal, meteorological factors, etc. factors have been met and even improved. We are confident that this achievement is motivated by technological advances. The quality of the final result and the effort that was required to obtain maps of this type less than a decade ago were not comparable to the current approaches (i.e., [32] where it was necessary to make interpolations for full generation of the cartography).

The volume of data produced by this experience is summarized in Table 5. The orthoimages covered almost 5 hectares and provided information at a resolution of 1.5 cm GSD.

**Table 5:** Volume of the biggest generated products.

Geomatic product	Size
Collected images	165 files 1.5 GB
Agisoft PhotoScan project	5.21 GB
Full orthoimage	488 MB
Nonground orthoimage	100 MB
Full orthoimage affection binary	26.3 MB
Nonground orthoimage affection binary	12.6 MB
Validation mask layer	7.22 MB

Some other intermediate products were also generated that required some extra space.

### Computer vision approach

**Fig 8:**
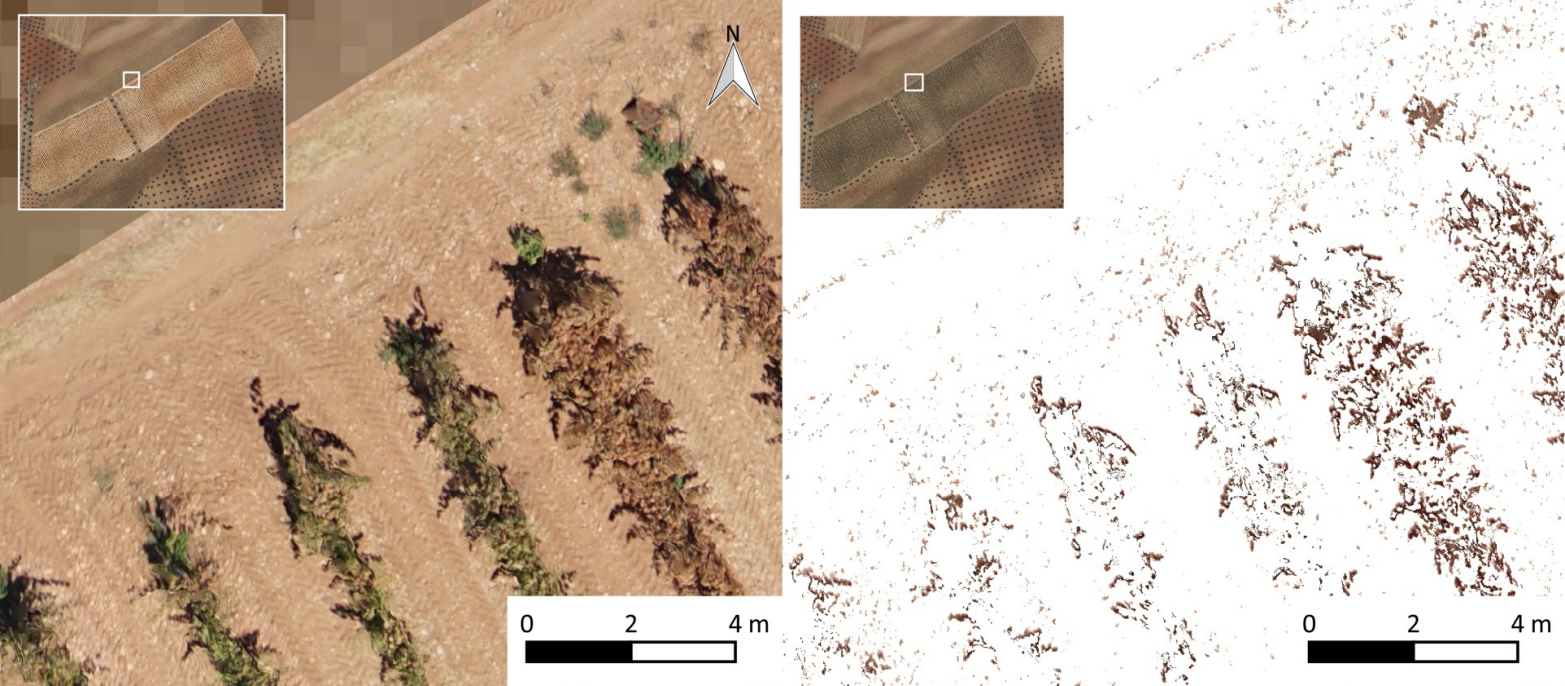
3D view of classified point cloud. Brown: ground. Green: medium vegetation. Pink: noise.

**Fig 9:**
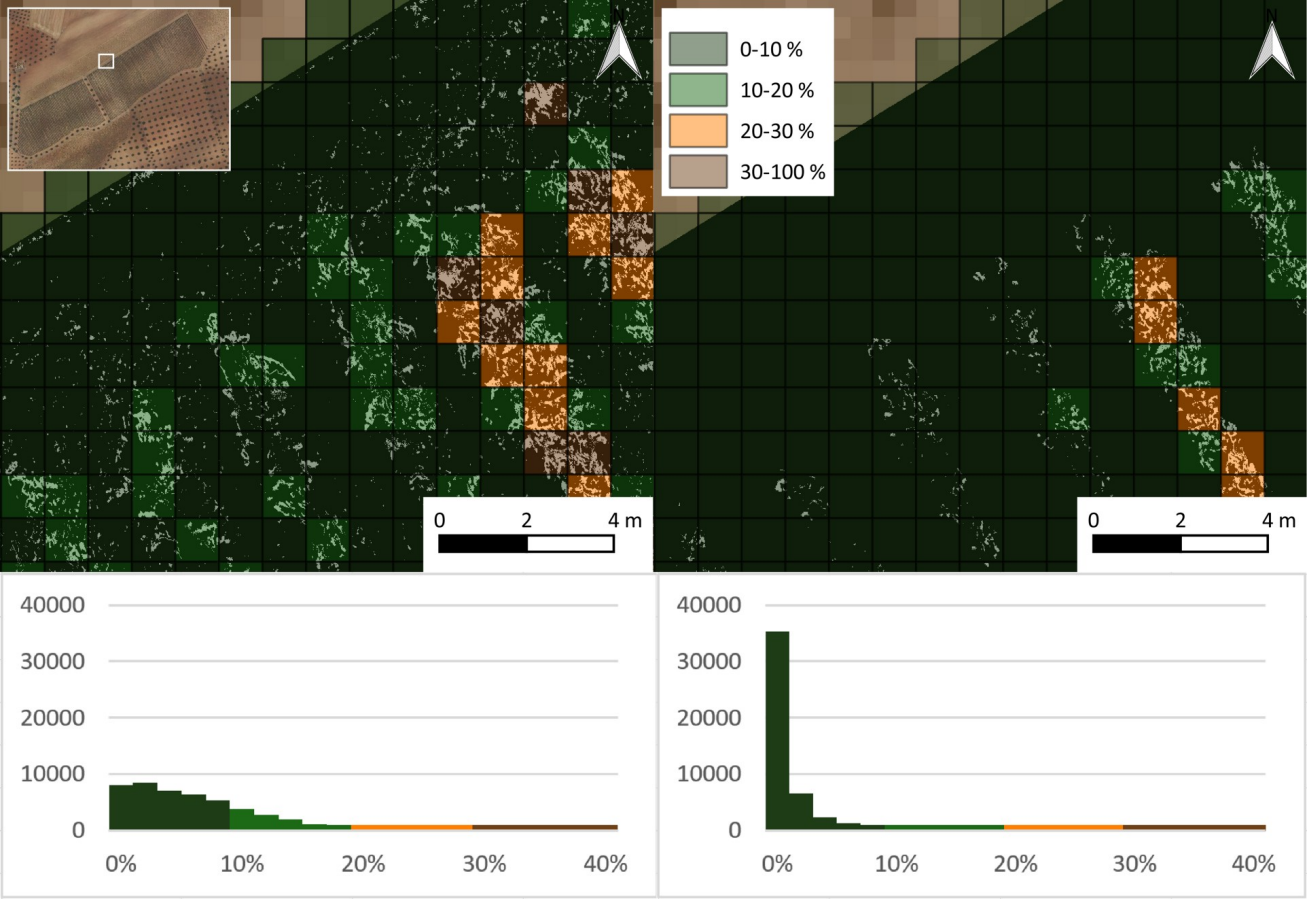
Orthoimage without ground (left) and impact detected in a nonground orthoimage (right).

**Fig 10:**
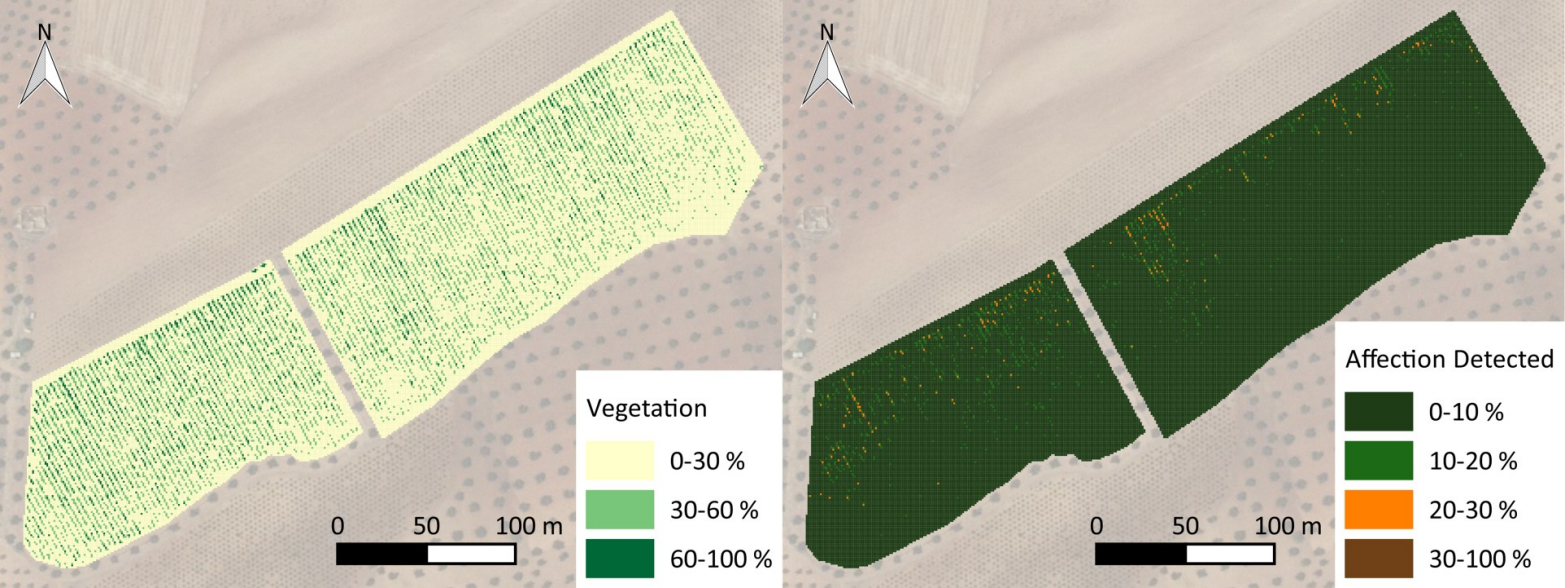
Maps of independent overall accuracies (up), independent commission error over the total considered pixels (center) and independent omission error over the total considered pixels (down) committed for both methodologies (full orthoimage left, nonground orthoimage right).

The results of affected vegetation segmentation from the entire orthoimage using computational vision techniques by the LAIC software [36] are shown in Fig 8. It should be noted, however, that there are several incorrectly detected pixels for affected vegetation over the entire orthoimage (ground, shadows, etc.). The radiometry of affected leaves is very similar to the radiometry of some parts of the soil; therefore, the ANN was unable to discriminate between these features. The problems from other studies revealed similar deficiencies. [30] and explained the obsolescence of traditional methods for remote sensing (such as satellites and conventionally piloted aircraft) compared to the response time and high resolution obtained with UAVs. Although [30] showed that UAVs can solve this problem, we detected a similar spectral response for the ground and affected vegetation, which indicates there is still a need for segmentation methodologies. These authors also concluded that it is important to divide the affection into different grades to perform adequate treatment of the differentiated areas. We divided the area into four affection grades, as shown in Fig 9 and Fig 10 on the right.

### Combination of geometric and computer vision techniques

**Fig 11:**
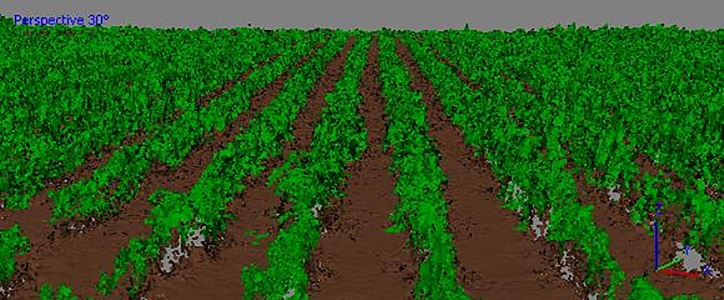
Percentage and histogram of impact detected on 1 m^2^ for the full (left) and nonground (right) orthoimages.

**Fig 12:**
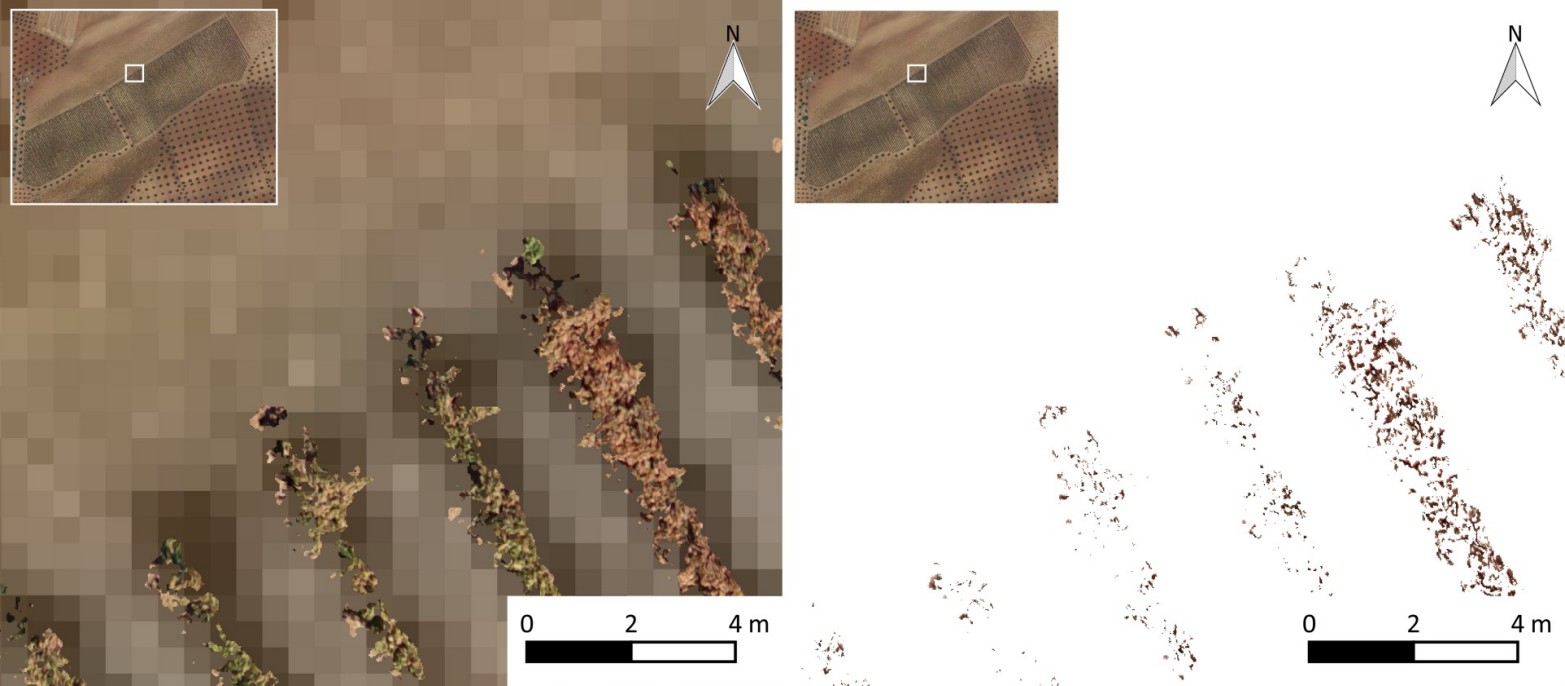
Maps of canopy cover (left) and the percentage of impact with the proposed methodology (right).

The dense point cloud generated in the photogrammetric process using the software Agisoft PhotoScan was classified according to the selected parameters for ground filtering. To this end, the ground was detected, and the remaining points were classified as medium vegetation (classification of LAS Specification Version 1.4 from the American Society for Photogrammetry & Remote Sensing). Dense point cloud results are shown in Fig 11. These results visually demonstrate good performance of the geometric algorithm for classification. A new orthoimage was generated by filtering vegetation points (Fig 12). The application of the previously calibrated ANN on this new orthoimage allowed for more accurate detection of affected vines (Fig 12). The detection over nonground orthoimage (Fig 12) showed better performance because of the detection of pixels located only where vegetation was present (not ground).

Other researchers also value the importance of considering full crop geometry with the third dimension (crop height) in the spatial analysis of crop monitoring because it is directly related to plant growth, biomass and yield quantification [18–22]. [20,21] used crop height to assess the pest affection as an indirect effect over biomass, but they did not directly detect the pest. However, no references have been found that utilize 3D information to better detect affection of pest in crops.

### Validation procedure

The analysis of validation comprised a total of 43,110,888 pixels. The confusion matrix, overall accuracy and success percentage of the pest segmentation following the proposed technique proposed are shown in Table 6 and Table 7.

**Table 6:** Confusion matrix of both proposed approaches.

		Validation mask		
		Affected	Unaffected	Total
**Full** **orthoimage**	**Affected**	313,358	2,414,547	2,727,905
	**Unaffected**	169,507	40,213,476	40,382,983
**Non-Ground** **orthoimage**	**Affected**	396,329	224,395	620,724
	**Unaffected**	86,536	42,403,628	42,490,164

**Table 7:** Success percentage tables for both proposed approaches.

	SPA	SPN
**Full orthoimage**	64.9%	94.3%
**Non-Ground orthoimage**	82.1%	99.5%

The overall accuracy (Eq 1) for the radiometric treatment was 94.0%, and the overall accuracy was 99.3% for the combination of radiometric and geometric treatments. Although we improved the OA through the proposed methodology, which reached almost 100%, if we only considered the result of this index, both methodologies appeared to detect the pest with high accuracy. However, it should be considered that only a small region of the area was covered by the crop (approximately 14% in this case). Thus, a deeper analysis of the performance of the methodologies over the crop was performed using the ratios described in the methodology (Table 7).

In comparing radiometric treatment (full orthoimage) to its combination with the geometric treatment (nonground orthoimage) of the geomatic information, the performance measured for the detection of affection through SPA (Eq 2) and SPN (Eq 3) improved the method (SPA 64.9% increased to 82.1% and SPN 94.3% to 99.5%) (Table 7).

The main problem occurred with the pixels that were determined to be affected by the analyzed approaches that were actually unaffected pixels, which represented 2,414,547 pixels for the full orthoimage and was reduced to 224,395 pixels (reduction of 91% of error by commission) after considering geometric and computer vision techniques. This problem was primarily produced by confusing affected vegetation with soil, which occurred more often in the approach with the full orthoimage than the approach that used the nonground orthoimage.

The spatial analysis of the committed errors is represented in Fig 7, in which it is possible to observe the affected areas that were more susceptible to incorrect detection.

As seen in Fig 7, the higher errors are located in the northern region of the crop and generally over the southwestern subarea as well. This result could have been caused by deficiencies in the irrigation system, which were communicated by the farmer. These irrigation deficiencies caused heterogeneity in plant vigor conditions and, therefore, in the level of affected vegetation because the affection was more intense in areas with higher vigor.

### Generation of affected area maps

Due to the performance of each methodology, the percentages of affected areas in the whole crop with both methods were calculated to show the differences between the methodologies in determining the affected vegetation for the whole parcel. The percentage of impact was calculated through both methodologies on 1 m^2^ cells of a vector grid (Fig 9) over the whole area. A color scale was applied to generate maps that allowed for the interpretation of the pest impact. Additionally, a map of the canopy cover was generated to compared the vegetation vigor to pest impact.

The affected area calculated for the entire plot was 6.43% with the full orthoimage and computer vision techniques, and it was 1.32% with a combination of the geometric and computer vision techniques. The average crop canopy cover in the plot was 14.2%, which meant that with the computer vision technique, the percentage of the affected crop was close to 45%, whereas the percentage obtained with the proposed methodology was close to 9%. A similar spectral response in the visible range of the ground and affected vegetation (brown color) produced an overestimation of impact that was close to four times larger. This overestimation is more evident for crops that do not have high green canopy cover values, i.e., woody crops.

The histogram in Fig 9 shows that the impact on the crop was dispersed with low frequencies of high-percentage impacts and vice versa. However, the percentage of impact was much higher in areas with high canopy cover than in areas with low canopy cover, as shown in Fig 10. Higher green canopy cover values in the northern region of the plot could be explained due to the inappropriate hydraulic design of the irrigation system. A manifold pipe located at the northern region of the plot was perpendicular to the trellis. Lateral pipes, which were larger than 120 m, resulted in low pressure, and therefore low discharge occurred at the end of the lateral pipes. Thus, vegetation growth was less vigorous at this location.

Although information from other spectral bands can add significant information to the health status of vegetation, the use of these cameras to detect the impact of the pest could lead to the same limitations of those described for RGB cameras if the soil and affected vegetation have a similar spectral response. Furthermore, the geometric characterization of the vegetation using these specific cameras could be less accurate than the characterization performed by RGB cameras, which was primarily due to its poor geometric resolution. Additionally, the economic cost of this equipment was much higher compared with RGB cameras. There is also more complexity in the algorithms to process that type of information [19,25,44].

In [33], a comparison between aerial hyperspectral and multispectral imaging techniques to detect citrus greening disease was conducted. The final conclusions were that there were errors in the geo-referencing, in the spectral purity of the values of the vegetation pixels, and in the atmospheric corrections as well as the variance and normalization of the illumination that could have biased the analysis. These effects were minimized when using RGB cameras-

## Conclusion

Conventional RGB cameras mounted on UAV platforms can be considered a very useful tool for pest aerial detection and quantification. Nevertheless, the enormous amount of information generated as a result of the photogrammetric workflow, i.e., 3D data, may be underused. Most users of UAV platforms are largely focused on the exploitation of 2D geomatic products. However, appropriately processed 3D products, such as accurate and classified points clouds, may improve the accuracy and utility of final applications, such as thematic maps. Compared to the 2D products, the 3D products incorporate the third dimension of a crop (height of the plant and orography), and they demonstrate an improvement in crop health characterization. In addition, incorporating the analysis of 3D information could solve soil distortions derived from remote-sensing techniques.

This study demonstrates that the combined use of computer vision and geometric techniques can enhance results through a proper clustering of the affected pixels. Furthermore, it can be concluded that a major source of error comes from similar radiometric responses to soil and affected vegetation for 2D products. This limitation is shared by consumer-grade cameras and by expensive thermal, multispectral and hyperspectral cameras. Computer vision techniques applied after soil segmentation will allow for more accurate detection of affected vegetation with low-cost RGB cameras mounted on UAVs.
